# Arbutin ameliorated ulcerative colitis of mice induced by dextran sodium sulfate (DSS)

**DOI:** 10.1080/21655979.2021.2005746

**Published:** 2022-01-03

**Authors:** Chuan Zhang, Huiping Zhu, Hui Jie, Hengyue Ding, Hongwen Sun

**Affiliations:** Department of Gastroenterology, Suzhou Traditional Chinese Medicine Hospital Affiliated College of Nanjing University of Chinese Medicine, Suzhou, Jiangsu, China

**Keywords:** Arbutin, ulcerative colitis, inflammation, apoptosis, JNK, p38

## Abstract

Accumulating evidence has revealed the anti-inflammatory effects of arbutin against various diseases. However, the effects of arbutin are not clarified in ulcerative colitis. This study was intended to investigate the protective effects and mechanisms of arbutin on DSS-induced colitis. Hematoxylin eosin staining was performed to determine the pathological damage of intestinal tissue in mice. Inflammatory factors levels in intestinal tissue were detected by enzyme linked immunosorbent assay (ELISA) assay. TUNEL staining showed the apoptosis levels of cells. Intestinal permeability was analyzed using the application of Fluorescein isothiocyanate Dextran (FD) 4. The levels of Zona Occludens 1 (ZO-1), occluding and claudin-1, and the related proteins in MAPK/ELK1 pathway were analyzed by Western blot. DSS promotes pathological injury, the levels of pro-inflammatory factors containing tumor necrosis factor alpha (TNF-α), Interleukin- 6 (IL-6) and myeloperoxidase (MPO), and cell apoptosis in the mouse colon. Additionally, intestinal permeability was increased and the levels of tight function-related proteins were increased following DSS induction. Its effects could be greatly improved by arbutin. Arbutin exerted effects by eliciting anti-inflammatory effects and maintaining normal intestinal mucosal barrier function, the action mechanism of which could be associated with MAPK/ELK1 pathway.

## Introduction

Ulcerative colitis (UC), which has been recognized as one of the modern inflammatory diseases [1], is a chronic, nonspecific inflammatory bowel disease (IBD) characterized by abdominal pain, diarrhea, bloody pus in the stools [2–6]. At present, it is believed that the treatment methods for UC mainly focus on reducing inflammatory response, promoting intestinal mucosal repair and improving intestinal mucosal barrier. The infiltration of a large number of inflammatory cells into the intestinal tract and their release of inflammatory factors will cause intestinal epithelial cell necrosis and shedding, increase intestinal mucosal permeability, thus leading to impaired intestinal mucosal barrier function [7]. MAPK signaling including JNK and p38 plays a vital role in UC, which can modulate NF-κB and c-Jun signaling pathways and is engaged in inflammation in UA [8,9]. A study reveals that mitogen-activated protein kinase(MAPK)-Elk-1 pathway is involved in the pathogenesis of UC induced by dextran sulfate sodium salt (DSS) and modulating expression of inflammatory factor [10].

Arbutin, a kind of natural antioxidant found mainly in bearberry leaf, has anti-inflammatory, antioxidative, antibacterial, hypoglycemic, anti-tumor and other pharmacological effects. A study has reported that arbutin has a protective effect on ethanol and aspirin-induced gastric ulcer animal models by regulating cytokine levels [1]. Recent studies have also shown that arbutin can increase the cell viability of intestinal epithelial cells [11]. It has been found that arbutin can act as a hydroxyl radical scavenging agent and inhibit the activation of p-JNK and p38 MAPK signaling proteins [12], and inhibition of p-JNK and p38 MAPK signaling can reduce the damage of DSS-induced UC in mice [9]. We assume that arbutin is able to improve DDS-induced UC and its mechanism could be related to MAPK/Elk-1 pathway. In this study, the purpose of this study was to explore whether arbutin can alleviate the inflammatory injury and intestinal epithelial cell apoptosis in UC and its mechanism in alleviating the epithelial dysfunction, thereby illustrating its effects and mechanism in UC.

## Method

### Animals

Forty male BALB/c mice with body weight of 16~20 g were fed adaptively for 1 week and randomly divided into control, DSS, Arbutin 50 mg/kg and Arbutin 100 mg/kg groups, with 10 mice in each group. Except for control group, UC model was established in other groups. Mice were gavaged with 3% DSS solution (DSS powder dissolved into sterile water) at a volume of 15 mL/kg continuously for 7 days. In DSS + Arbutin group, the mice were administrated with abutin 50 mg/kg and 100 mg/kg for 7 days by intragastric administration, once a day. The mice were sacrificed by cervical dislocation. The study was approved by the ethical committee of Suzhou Traditional Chinese medicine Hospital Affiliated College of Nanjing University of Chinese Medicine.

### Disease activity index (DAI)

General situation and UC disease activity index: including mouse behavior, body weight, food consumption and evaluation of DAI based on visual observation of stool. Scoring criteria are as follows: 0 points: large stool – normal, cryptic blood or blood stool – negative; 1 point: stool trait – soft stool, occulted blood or bloody stool – negative; 2 points: stool trait – soft stool, occult blood or blood stool – positive occult blood; 3 points: positive fecal traits – diarrhea, occult blood or bloody stools – occult blood; 4 points: large stool trait – diarrhea, occulted blood or bloody stool – bloody stool.

### HE staining

In each experiment, 10 samples of target colon tissue were randomly selected and cut along the longitudinal axis. The sections were usually fixed and stained with hematoxylin-eosin. The histopathological changes of the colon, including the size and number of ulcers and the changes of epithelial hyperplasia, were observed under an ordinary light microscope. Histopathological score was determined by a previous report [13].

### ELISA assay

The levels of TNF-α, IL-6 and IL-10, together with MPO in intestinal tissue in all experimental groups were determined by ELISA kits (TNF-α kit, Shanghai Jianglai industrial Limited By Share Ltd., Shanghai, China. Mouse IL-6 ELISA Kit, ab100713; Mouse IL-10 ELISA kit, ab108870; abcam, England. MPO ELISA Kit, SP14386, Wuhan saipei Biotechnology Co., Ltd., Wuhan, China), respectively. The procedure was performed strictly according to the instructions of the kit.

### Western blot

The colon tissue of mice was analyzed with RIPA lysate (ThermoFisher Inc.). Total protein in the supernatant collected was quantified by BCA method. Total protein was then subjected to SDS agarose gel electrophoresis and then transferred into PVDF membrane. The primary antibodies (Abcam, England) were added and incubated with bands overnight at 4°C. Following TBST washing, the protein bands were then incubated with the secondary antibody (Abcam, England) at room temperature for 2 h. Then, the ECL chemiluminescence reagent (ThermoFisher Inc.) was used for color development and chemiluminescence imaging.

### Intestinal Permeability Method

The mice were anesthetized by isoflurane inhalation. The 5 cm intestinal segment with rich mesangial blood vessels was selected and ligated with silk thread at both ends. One end of the free intestinal segment was loosely ligated and 0.5 mL Fluorescein isothiocyanate Dextran (FD4) solution (10 mg/ mL) was extracted with a 1 mL syringe and injected into the intestine. After 30 min, FD4 in intestinal lumen entered the blood circulation through the mesenteric vessels. The mice were sacrificed by cervical dislocation and blood samples were taken from the hearts of the mice. The blood was centrifuged at 10,000 g at 4°C for 10 min, and the concentration of FD4 (Sigma) was measured using a fluorospectro photometer (Thermo Scientific Lumina).

### RT-qPCR

Total RNA in colon tissue was extracted with Trizol reagent (Takara, Japan), and reverse transcription was performed according to the instructions of the reverse transcription kit (Transcriptor First Strand cDNA Synthesis Kit, Roche). cDNA was quantified with SYBR®Green Realtime PCR Master Mix-Plus (TOYOBO, Japan). GAPDH was used as an internal reference. The ratio of mRNA relative to the control group was calculated by 2^−Δ Δ CT^ method. The primers in this study are as following: zonula occludens (ZO)-1, Forward: 5ʹ- GCCGCTAAGAGCACAGCAA-3ʹ, Reverse: 5ʹ-GCCCTCCTTTTAACACATCAGA-3ʹ; occludin, Forward: 5ʹ- TGAAAGTCCACCTCCTTACAGA −3ʹ, Reverse: 5ʹ- CCGGATAAAAAGAGTACGCTGG −3ʹ; claudin-1, Forward: 5ʹ- TGCCCCAGTGGAAGATTTACT-3ʹ, Reverse: 5ʹ- CTTTGCGAAACGCAGGACAT-3ʹ; GAPDH, Forward: 5ʹ- AGGTCGGTGTGAACGGATTTG −3ʹ, Reverse: 5ʹ- GGGGTCGTTGATGGCAACA −3ʹ.

### Statistical analysis

Graph 7.0 was used for statistical analysis. The experimental data were presented as mean ± standard deviation (mean ± SD). One-way analysis of variance was used for comparison of parametric data of among groups, followed by Turkey’s test for comparison between two groups, while Mann-Whitney *U* test was performed for difference analysis of non-parametric data, and *P < *0.05 indicates that the difference is statistically significant.

## Results

### Administration of arbutin significantly improved UC symptoms in mice

In our study, mice were administrated with 2.5% DSS in water for 7 days to induce UC, an acute inflammatory model. During the experiment, mice in control group remained a normal state, while those in Model group mice presented fewer appetites, hair miscellaneous disorder, poor mental state, persistent loose bleeding and decreased body mass, which was consistent with the reports by previous literature.

Effect of the arbutin on DSS-induced colitis was analyzed by the body weight loss, DAI score, histopathological analysis of colon tissue and colon length.

**Figure 1.**
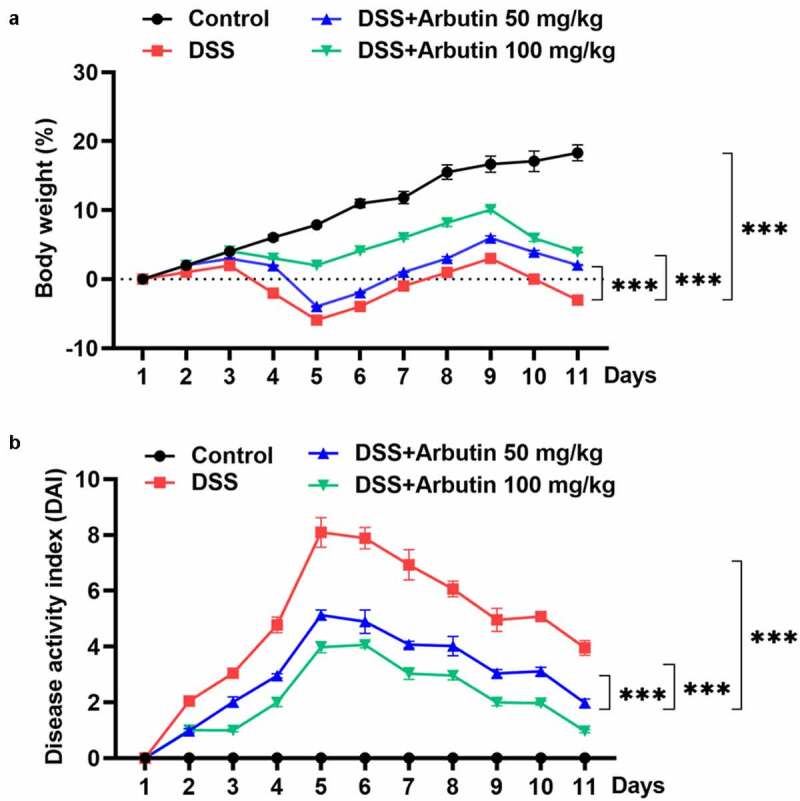
Arbutin administration reduced body weight and DAI score. Mice in DSS group were gavaged with 3% DSS solution at a volume of 15 mL/kg continuously for 7 days and the mice in DSS + Arbutin group were also administrated with abutin 50 mg/kg or 100 mg/kg for 7 days by intragastric administration, once a day. Body weight (a) and DAI score (b) were recorded every day. The values presented are mean ± SD (*n* = 10/each group). ***P < 0.001

**Figure 2.**
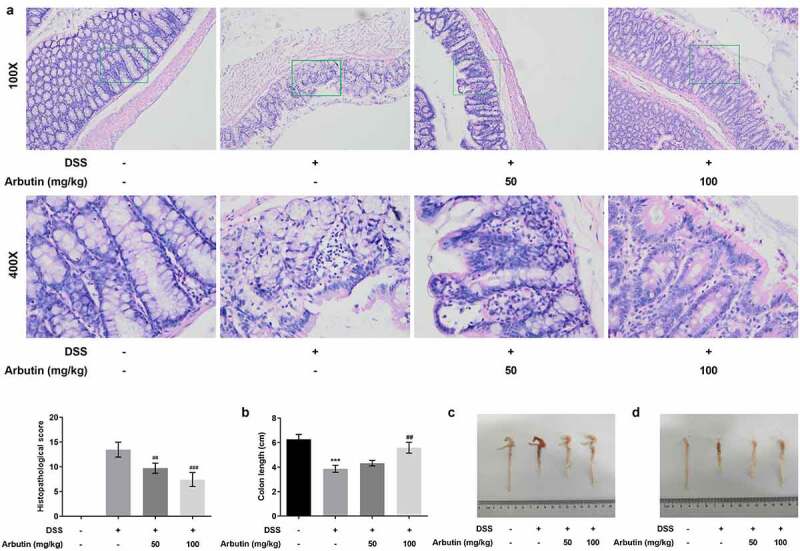
Arbutin improved intestinal injury and increased colon length. Intestinal histopathology (a) was observed in each group through HE staining. The colon length (b). The colon is removed and photographed (c, d). The data were shown in mean ± SD (*n* = 10/each group). ***P < 0.001 versus DSS (-). ^##^*P < *0.01 versus DSS (+) + Arbutin (-)

To evaluate the effect of arbutin on DSS-induced UC, the body weight and DAI score of mice in each group were examined, along with the histopathological analysis of colon tissue and colon length. Notably, body weight of mice in control group was rising steadily. However, from day 3 to day 5, it presented a descended trend, which was then recovered gradually in DSS group. Arbutin administration improved the body weight loss, especially at the dose of 100 mg/kg (Figure 1(a)). As compared with the control group, DAI score in DSS group was increased obviously from day 1. Arbutin treatment groups showed a more significant decrease than that observed in the DSS group on day 5–11 (Figure 1(b)). As can be seen from the results in Figure 1(c), no ulcer was found in the colon tissue of the blank group, and the glands were neatly arranged and the mucosa was complete. In the model group, the colonic mucosa was defective, glandular structure separated, and a large number of neutrophils were infiltrated. Furthermore, we observed visible inflammatory reaction, granulation tissue hyperplasia and fibrosis, and obvious lesions deeply concentrated in the muscular layer and the serosal layer. It is suggested that this model has caused deep mucosal injury associated with inflammation in the colon. Compared with the model group, the histopathological observation of arbutin group was improved to varying degrees (Figure 2(a)). Additionally, arbutin markedly suppressed colon shortening when compared with that observed in DSS-treated group (Figure 2(b–d)).

### Arbutin affected the expression of inflammatory factors and decreased apoptotic levels

**Figure 3.**
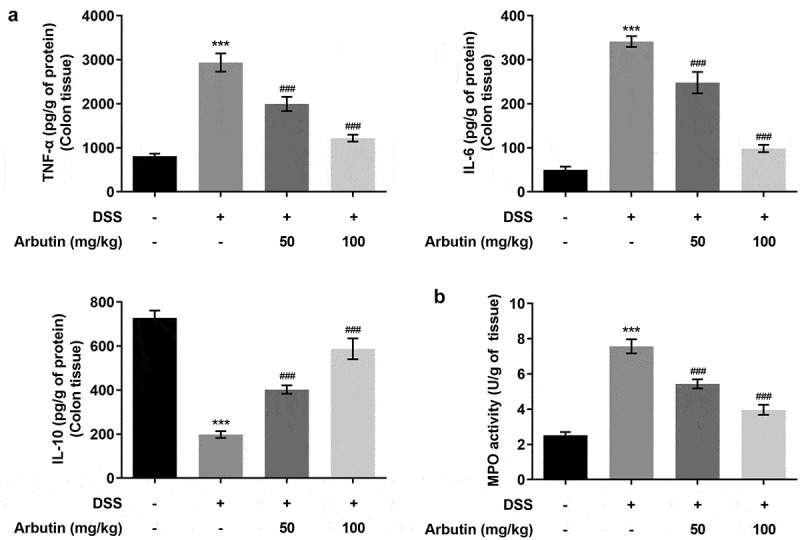
Arbutin altered the levels of inflammatory factors of colon tissue. The detection of inflammation-related factors(a). MPO activity (b). The data were shown in mean ± SD (*n* = 10/each group). ***P < 0.001 versus DSS (–). ^###^*P < *0.001 versus DSS (+) + Arbutin (–)

**Figure 4.**
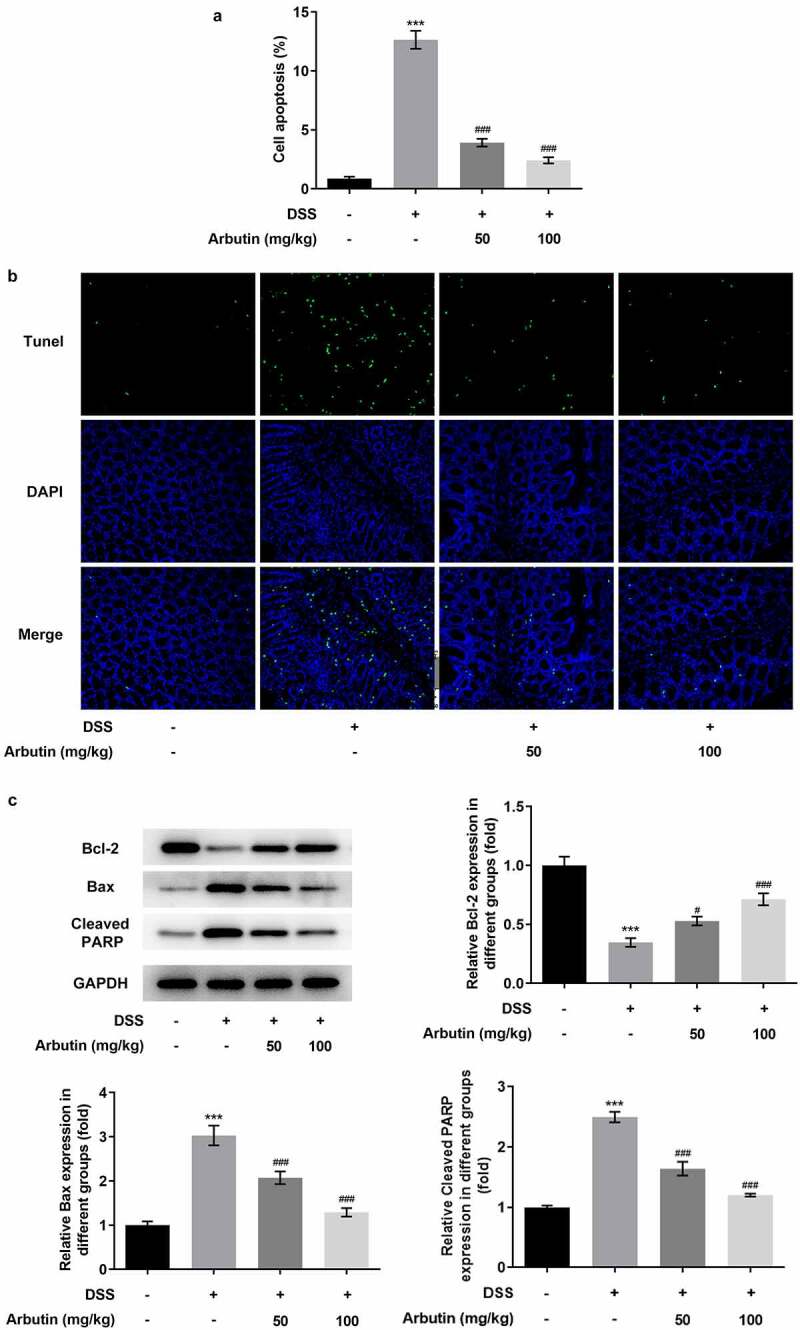
Arbutin decreased apoptosis levels of colon tissue. Tunel staining (a, b). The detection of apoptosis-related proteins (c). The data were presented in mean ± SD (*n* = 10/each group). ***P < 0.001 versus DSS (–). ^#^*P < *0.05, ^###^*P < *0.001 versus DSS (+) + Arbutin (–)

To evaluate the effects of arbutin on inflammation and apoptosis, we determined the levels of inflammatory mediators, TNF-α and IL-6 showing that α diversity was increased in model group compared with control group, while IL-10 showed a decrease (Figure 3(a)). Administration of arbutin reduced the relative levels of TNF-α and IL-6 and increased IL-10 levels (Figure 3(a)). Compared with control group, the activity of MPO in DSS group was significantly increased. MPO activity in arbutin-treated group was significantly decreased (Figure 3(b)). Increased apoptosis is also one of the causes of intestinal tissue damage and decreased mucosal barrier function. Then, the level of apoptosis in colon of mice was measured through TUNEL staining. The apoptosis level in DSS group was increased significantly compared with control group. However, treatment of arbutin contributed to restoring the apoptosis to a normal level when compared with the model group (Figure 4(a,b)). In addition, arbutin administration resulted in dramatic decreases in the Bax, cleaved PARP levels compared to that of DSS group in a dose-dependent manner. However, the level of Bcl2 in Arbutin group was significantly lower than that of model group (Figure 4(c)).

Arbutin alleviates DSS-induced dysfunction of intestinal epithelial cells and modulates MAPK/ELK1 pathway

**Figure 5.**
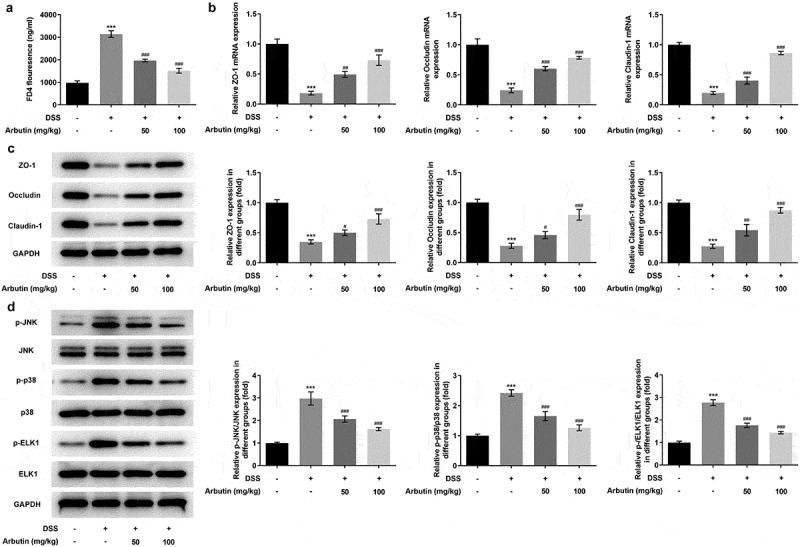
Arbutin increased the expression levels of tight junction proteins and affected MAPK/ELK1. FD4 fluorescence (a), the detection of tight junction proteins through Western blot and RT-qPCR (b, c), the detection of MAPK/ELK1 signals (d). The data were presented in mean ± SD (*n* = 10/each group). ***P < 0.001 versus DSS (–). ^#^*P < *0.05, ^##^*P < *0.01, ^###^*P < *0.001 versus DSS (+) + Arbutin (–)

In order to determine the effects of arbutin on ileum permeability and reveal the molecular mechanisms of arbutin improving DSS-induced colon injury, the permeability of ileum to FD4 was determined. The results showed that compared with control group, the ileum permeability of DSS mice to FD4 was significantly increased, while arbutin treatment markedly reduced its permeability when compared with DSS group (Figure 5(a)). The expression of tight junction protein ZO-1, Occludin and Claudin-1 in ileum was detected by Western blot. Arbutin administration resulted in dramatic increases in the ZO-1, occluding and claudin-1 compared to DSS group (Figure 5(b,c)). Subsequently, the purpose of this study was to investigate whether arbutin can affect the expression of MAPK/ELK1 signaling protein in DSS-induced UC. The phosphorylation levels of p-JNK, p-p38, p-ELK1 in DSS-induced mice were significantly higher than the control group. Arbutin could reverse the levels of p-JNK, p-p38, p-ELK1 in UC mice (Figure 5(d)).

## Discussion

In this study, we attempted to identify the effect and mechanism of arbutin by conducting a series of experiments in DSS-induced mice. DDS administration led to pathological inflammation and apoptosis in the colon tissues of experimental animals, and induced intestinal mucosal barrier dysfunction, while arbutin could improve intestinal injury caused by DSS. In addition, we found that DSS group exhibited aggravated pathological injury of intestinal tissue and decreased colon length, which could be significantly recovered by arbutin treatment. Additionally, TNF-α and IL-6 levels were decreased, and IL-10 was decreased in colon tissue of DSS-induced mice compared with control group. Oral administration of arbutin, however, could markedly reverse these effects. The mechanism by which IL-6 knockout can decrease the increase of intestinal epithelial permeability may be related to the increase of IL-10 level [14]. TNF-α, a key mediator of the inflammatory status in various diseases, can increase the permeability of intestinal epithelial cells in vitro [15,16]. Besides, the increased levels of pro-inflammatory cytokines are related to the severity of experimental colitis [17]. Therefore, arbutin could improve intestinal epithelial permeability through affecting inflammatory factor levels. We further found that arbutin treatment decreased the apoptosis levels of cells in the intestinal tissue of DSS-induced mice, increased Bcl-2 level and decreased the levels of Bax and cleaved PARP. These results demonstrated that arbutin suppressed the apoptosis induced by DSS possibly through regulating the levels of these apoptosis-related proteins.

Arbutin had an effect on ameliorating the ileum permeability and increasing the levels of tight junction proteins, Occludin, Claudin and ZO-1 diversity. Mechanical barrier is the main component of intestinal mucosal barrier, which is composed of intestinal epithelial cells and the connections between cells. Among the wide range of connections in the mechanical barrier, tight junction, composed mainly of tight junction proteins, including Occludin, Claudin and ZO-1, plays a significant role [18,[19]. The integrity of intestinal epithelial tight junctions, however, can be destroyed by inflammatory cytokines, leading to impaired normal intestinal mucosal barrier function [20–22]. Collectively, arbutin could maintain intact tight junction morphology and function. Western blot analysis carried out in this study revealed obvious differences in the phosphorylation level of JNK, p38 and ELK1 in colon tissue of DSS-induced compared with that in control group. The modulation of p38/JNK signaling involved the regulation of inflammation and apoptosis [23–26]. Furthermore, accumulating evidence revealed the implication of MAPK signaling in the mechanism of colitis [9,27,28]. A study reported that the induction of Elk-1 transcriptional activity could be mediated through either the synergistic efforts of JNK and p38 MAP kinases or JNK alone [29]. Therefore, the action mechanism of arbutin involving in inflammation, apoptosis and normal intestinal mucosal barrier function in UC induced by DSS could be related to MAPK/ELK1 signaling pathway.

## Conclusion

This study demonstrated that arbutin exerted protective effects on UC mice by regulating inflammation, apoptosis and intestinal mucosal barrier function, which could be mediated by MAPK/ELK1 signaling pathway. Arbutin could be a potential drug for US management, which requires deeper investigations in clinical practice.

## Data Availability

The datasets used and/or analyzed during the current study are available from the corresponding author on reasonable request.

## Highlights

1. Arbutin ameliorates the pathological injury caused by dextran sodium sulfate.

2. The protective mechanism of arbutin is involves in inflammation and apoptosis.

3. Arbutin improves the dysfunction of intestinal epithelial cells.

4. Arbutin exerts a protective role possibly mediated by MAPK/ELK1 pathway.
